# Real-Time Monitoring of Dissection Events of Single Budding Yeast in a Microfluidic Cell-Culturing Device Integrated With Electrical Impedance Biosensor

**DOI:** 10.3389/fbioe.2021.783428

**Published:** 2021-10-27

**Authors:** Zhen Zhu, Yangye Geng, Yingying Wang, Ke Liu, Zhenxiang Yi, Xiangwei Zhao, Shuiping Ouyang, Ke Zheng, Yimin Fan, Zixin Wang

**Affiliations:** ^1^ Key Laboratory of MEMS of Ministry of Education, Southeast University, Nanjing, China; ^2^ State Key Laboratory of Bioelectronics, Southeast University, Nanjing, China; ^3^ College of Chemical Engineering, Nanjing Forestry University, Nanjing, China; ^4^ School of Electronics and Information Technology, Sun Yat-Sen University, Guangzhou, China

**Keywords:** electrical impedance spectroscopy (EIS), microfluidic biosensor, single-cell analysis (SCA), yeast aging, replicative lifespan, dissection event

## Abstract

Microfluidic devices in combination with fluorescent microscopy offer high-resolution and high-content platforms to study single-cell morphology, behavior and dynamic process in replicative aging of budding yeast, *Saccharomyces cerevisiae*. However, a huge mass of recorded images makes the data processing labor-intensive and time-consuming to determine yeast replicative lifespan (RLS), a primary criterion in yeast aging. To address this limitation and pursue label-free RLS assays, electrical impedance spectroscopy (EIS) that can be easily functionalized through microelectrodes in microfluidic devices, was introduced to monitor cell growth and division of budding yeast. Herein, a microfluidic device integrated with EIS biosensor was proposed to perform *in-situ* impedance measurement of yeast proliferation in single-cell resolution so as to identify the momentary events of daughter dissection from its mother. Single yeast cells were reliably immobilized at the bottleneck-like traps for continuous culturing, during which daughter cells were effectively detached from their mother cells by hydraulic shear forces. Time-lapse impedance measurement was performed every 2 min to monitor the cellular process including budding, division and dissection. By using the K-means clustering algorithm to analyze a self-defined parameter “*Dissection Indicator*,” to our knowledge for the first time, the momentary event of a daughter removing from its mother cell was accurately extracted from EIS signals. Thus, the identification of daughter dissection events based on impedance sensing technology has been validated. With further development, this microfluidic device integrated with electrical impedance biosensor holds promising applications in high-throughput, real-time and label-free analysis of budding yeast aging and RLS.

## Introduction

Budding yeast, *Saccharomyces cerevisiae* (*S. cerevisiae*), benefiting from its fast proliferation, short lifespan, easy maintenance, and fully-sequenced genome, has been extensively used as a model organism in aging studies (Gershon and Gershon, 2000; Fontana et al., 2010; Lippuner et al., 2014). Replicative lifespan (RLS) of budding yeast, defined as the number of daughter cells that a mother cell produces before it ceases to divide, has been widely investigated since the conventional manual microdissection method was proposed in 1959 (Mortimer and Johnston, 1959).

In recent years, a variety of microfluidic devices featuring micron-scaled shallow cavities (Fehrmann et al., 2013), slim channels (Li et al., 2017), compressing pads (Lee et al., 2012; Zhang et al., 2012; Huberts et al., 2013), or obstacle traps (Crane et al., 2014; Jo et al., 2015; Liu et al., 2015; Sarnoski et al., 2018), have emerged rapidly for yeast culturing and replicative aging studies. With the application of those devices in single-cell capturing, culturing and optical screening, RLS of budding yeast has been thus determined by analyzing massive time-lapse images. As such, intensive data acquisition and time-consuming image processing have put forward a major challenge to the real-time analysis of dynamic cellular process, especially for high-throughput and long-term single-cell replicative aging assay and RLS determination of budding yeast.

Electrical impedance spectroscopy (EIS), as an alternative to optical imaging, is a non-invasive, label-free and multiparametric tool to probe biophysical properties of biological specimens, such as cells and tissues (Morgan et al., 2007; Bürgel et al., 2016; Schmid et al., 2016; Xu et al., 2016). By integrating microelectrodes with cell traps in microfluidic devices, *in-situ* EIS measurement of immobilized cells enables continuous monitoring of dynamic cellular process in single-cell resolution over an extended time period, such as cell growth and motion (Zhu et al., 2014), mitosis and cytokinesis (Ghenim et al., 2010; Zhu et al., 2015), as well as cell differentiation (Zhou et al., 2016). Although diverse strategies to process the raw electrical impedance signals are efficient to elucidate morphological and phenotypical differences of cells, to the best of our knowledge, real-time monitoring of yeast daughter dissections using *in-situ* electrical impedance sensing technology has not yet been reported. Therefore, developing a label-free and non-optical method to identify the dissection events of yeast daughter cells is desirable.

In this work, we report on an EIS-biosensor-integrated microfluidic device, which is capable of hydrodynamic trapping, continuous culturing and *in-situ* electrical impedance monitoring of single budding yeast cells. The microfluidic device features: i) bottleneck-like traps for reliable capture, long-term retention and continuous culturing of yeast cells, as well as automatic and effective removal of daughter cells through hydraulic shear forces; and ii) localized coplanar microelectrode pairs for real-time and label-free detection of daughter-cell dissections using time-lapse *in-situ* electrical impedance biosensor. In addition, the K-means clustering algorithm was used to compensate any baseline drift in the recorded EIS signals and accurately extract momentary information of daughter-cell dissection events.

## Materials and Methods

### Device Design

**FIGURE 1:**
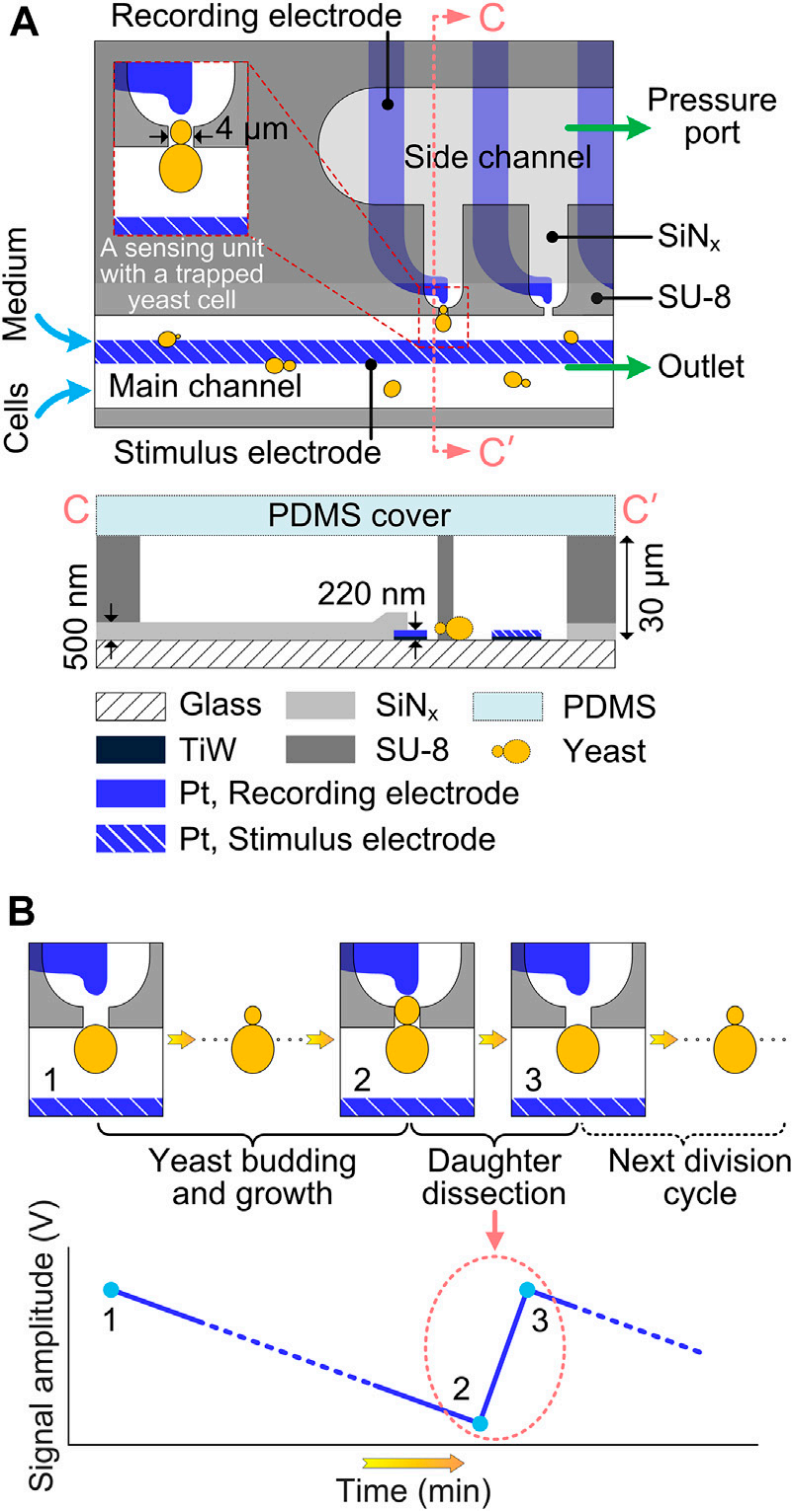
Schematics of EIS-biosensor-integrated microdevice for real-time monitoring of single budding yeast cells. **(A)** Top view and Cross-sectional view along CC′ in the impedance sensing area of the microfluidic device. Insert shows the impedance sensing unit with an immobilized budding yeast cell. For better illustration, dimensions are not to scale. **(B)** *In-situ* sensing principle of the yeast dissection event using the electrical impedance biosensor. The instantaneous detachment of daughter cell results in a drastic increase (from 2 to 3) of EIS signal amplitude.

Figure 1A schematically illustrates the microfluidic device for immobilization, culturing and *in-situ* impedance monitoring of single budding yeast cells. The microdevice consists of 5 layers, i.e., a glass substrate, Pt electrodes, a SiN_x_ passivation layer, SU-8 microfluidic channels and a polydimethylsiloxane (PDMS) cover. The SU-8 fluidic network comprises a main channel (width: 150 μm) and a parallel side channel (width: 300 μm), with 10 bottleneck-like traps (narrow neck width: 4 μm) connecting those two channels. Budding yeast cell suspension and culturing medium were infused into the main channel *via* their corresponding inlets (i.e., cell inlet and medium inlet, respectively), both at a flow rate of 1.5 μL/min. By precisely controlling the underpressure (about -6,000 Pa) applied to the pressure port, single yeast cells were reliably captured at the trap orifices. After cell immobilization, the pressure was raised to a constant value (about 2,000 Pa) to prevent redundant cells from being captured at the same traps. To integrate electrical impedance biosensor, Pt microelectrode pairs were patterned underneath the SU-8 structures, with the SiN_x_ passivation layer in between. To implement *in-situ* impedance measurement, long common electrode in the main channel serves as the stimulus electrode to supply an excitation alternating current (AC) voltage, and tip individual electrodes around trap orifices serve as recording electrodes to receive the weak response current, which is then amplified and converted into a detectable voltage signal. The SiN_x_ layer was reopened in the sensing region to reduce potential electric crosstalk between adjacent electrodes, and in the contact pads around the device border to connect electrodes to external circuits. Thus, the sensing unit, which features a trap for cell capture and retention and a pair of microelectrodes for *in-situ* electrical impedance measurement, is the core composition of the EIS-integrated microdevice.

To perform real-time monitoring of yeast dissection events by using *in-situ* electrical impedance biosensor, single cells are firstly immobilized at the traps and continuously cultured under laminar-perfused medium in the microfluidic device. During cell culturing for an extended time period, electrical impedance measurement is carried out every 2 min to monitor the dynamic cell process, such as budding, cytokinesis and daughter dissection. As soon as the cytokinesis-completed daughter cell is detached from its mother by hydraulic shear forces, it is immediately dragged away by the continuous medium flow, thereby suddenly cutting down the cell impedance between the stimulus and recording electrodes. This drastic decrease in impedance, corresponding to the momentary event of daughter dissection, can be detected by the time-lapse impedance measurement, showing a steep signal jump on the amplitude curve of EIS signal (Figure 1B).

### Device Fabrication

The fabrication process of the microfluidic device (Figure 1A) is shortly described here. First, 200-nm-thick Pt with a 20-nm-thick TiW seed layer were patterned on a 4-inch glass wafer using a lift-off process. Then, a 500-nm-thick SiN_x_ layer was deposited on the wafer by plasma-enhanced chemical vapor deposition (PECVD), followed by reactive ion etching (RIE) to define the opening in sensing region and contact pads. Subsequently, 30-μm-thick SU-8 3,025 photoresist (MicroChem, United States) was spin-coated on the wafer with patterned microelectrodes, and structured into microfluidic channels by using standard photolithography. After wafer dicing, an unstructured PDMS (Sylgard 184, Dow Corning, United States) layer, punched with holes as fluidic inlets and outlets was used to seal the microfluidic channel network. For an irreversible bond between PDMS and SU-8, both SU-8 surface of each die and oxygen-plasma-activated PDMS cover were modified with 3-aminopropyltriethoxysilane (APTES, Merck KGaA, Germany) in aqueous solution (Zhu et al., 2016).

### Experimental Setup

**FIGURE 2:**
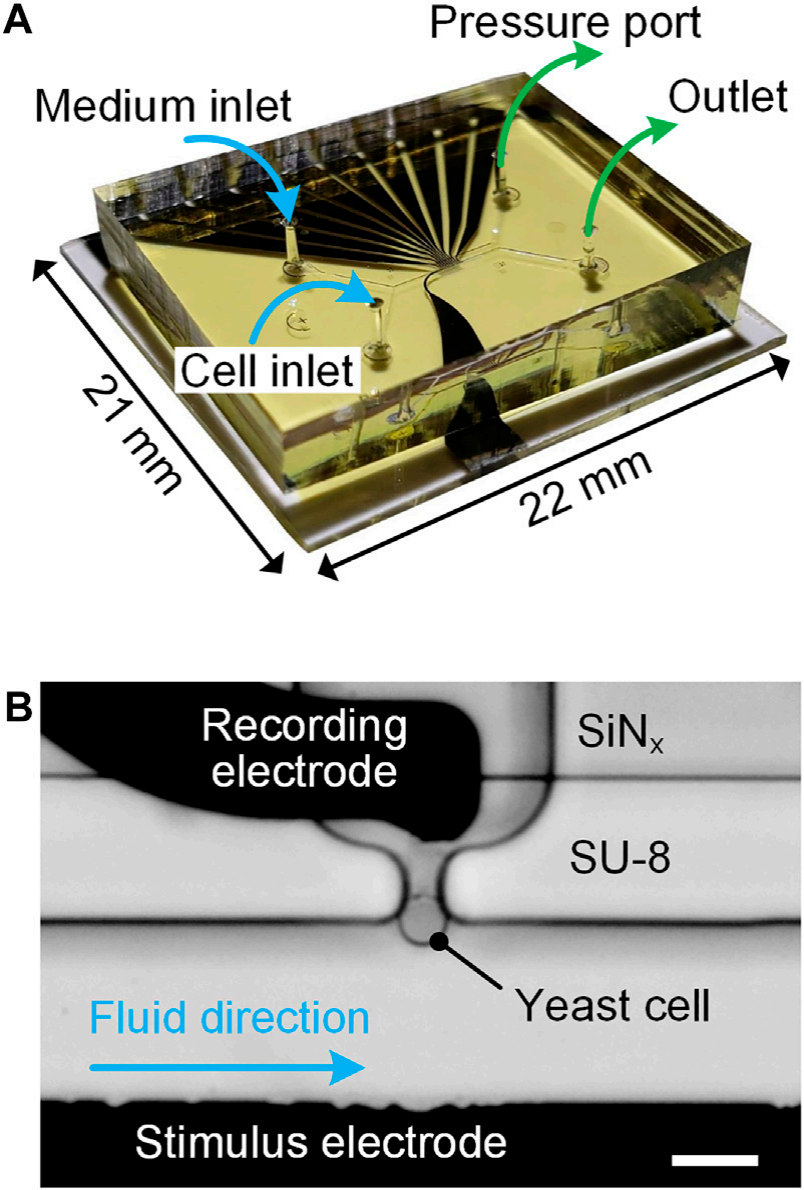
**(A)** Photograph of an assembled microfluidic device indicating fluidic connections for inlets and outlets. **(B)** Micrograph of a sensing unit: a bottleneck-like trap immobilized with a single yeast cell. Scale bar is 10 μm.

To achieve single-cell immobilization and real-time impedance measurement in a sensing unit, an experimental setup was designed and its details were presented in our previous work (Zhu et al., 2015). Briefly, a fabricated microfluidic device (Figure 2) was first clamped between a custom-made aluminum (Al) holder and a transparent polymethylmethacrylate (PMMA) cover by using screws. Then, a printed circuit board (PCB) soldered with spring probes was mounted on the PMMA cover to electrically connect microelectrodes with an impedance spectroscope (HF2IS, Zurich Instruments, Switzerland) and a current amplifier (HF2CA, Zurich Instruments, Switzerland). Two glass syringes, prefilled with cell suspension and culturing medium, were respectively connected to cell and medium inlets through polytetrafluoroethylene (PTFE) tubes for fluidic access. Both syringes were affixed on a precision syringe pump (neMESYS, Cetoni, Germany) for sample delivery. A pressure controller (OB1 MK3+, Elveflow, France) was used to adjust the underpressure applied to the pressure port for cell immobilization. For time-lapse imaging, the assembled setup, including the Al holder, microfluidic device, PMMA cover and PCB was placed on the stage of an inverted microscope (FV3000, Olympus, Japan).

For time-lapse EIS measurement, a swept-frequency AC voltage $V_{sti}^{*}$ (10 kHz–10 MHz, V_p_: 1 V) from the impedance spectroscope was applied to the stimulus electrode at an interval of 2 min, and the response current $I_{res}^{*}$ obtained from the corresponding recording electrode was amplified and converted into a voltage signal $V_{rec}^{*}$ using the current amplifier, and ultimately recorded by the impedance spectroscope. The relationship among the measured impedance $Z^{*}$ , the response current $I_{res}^{*}$ and the recorded voltage $V_{rec}^{*}$ can be expressed by following equations: $Z^{*}=V_{sti}^{*}/I_{res}^{*}$ , $V_{rec}^{*}=G\cdot I_{res}^{*}$ , and $Z^{*}=G\cdot V_{sti}^{*}/V_{rec}^{*}$ . Accordingly, the measured impedance $Z^{*}$ is inversely proportional to the response current $I_{res}^{*}$ ( $Z^{*}\propto 1/I_{res}^{*}$ ), and also inversely proportional to the recorded voltage $V_{rec}^{*}$ ( $Z^{*}\propto 1/I_{rec}^{*}$ and $\left|Z^{*}\right|=G/A$ , where A is the amplitude of $V_{rec}^{*}$ ). Thus, the EIS amplitude A can be directly used to characterize the impedance variation induced by the immobilized budding yeast cells.

### Cell Preparation

A budding yeast (*S. cerevisiae*) cell strain, derived from BY4743, was used in the experimental verification of the EIS-biosensor-integrated microfluidic device. Yeast cells were cultured using standard methods as described in literature (Sherman, 2002). Liquid cultures of yeast were grown at 30°C in a synthetic complete (SC) medium, which contained 0.17% yeast nitrogen base without amino acids or ammonium sulfate, 2% glucose, and a complement of amino acids and nucleotides. 0.05% w/v Pluronic^®^ F127 solution was added in the SC medium to prevent hydrophobic components from sticking together or accumulating on the channel surface. Prior to cell filling in the glass syringe, the suspension of yeast cells was diluted to reach a concentration of approximately 1 × 10^6^ cells/mL in SC medium. All chemicals were purchased from Merck, Germany.

## Results

### Continuous Culturing and Automatic Daughter Dissections of Single Budding Yeast Cells

**FIGURE 3:**
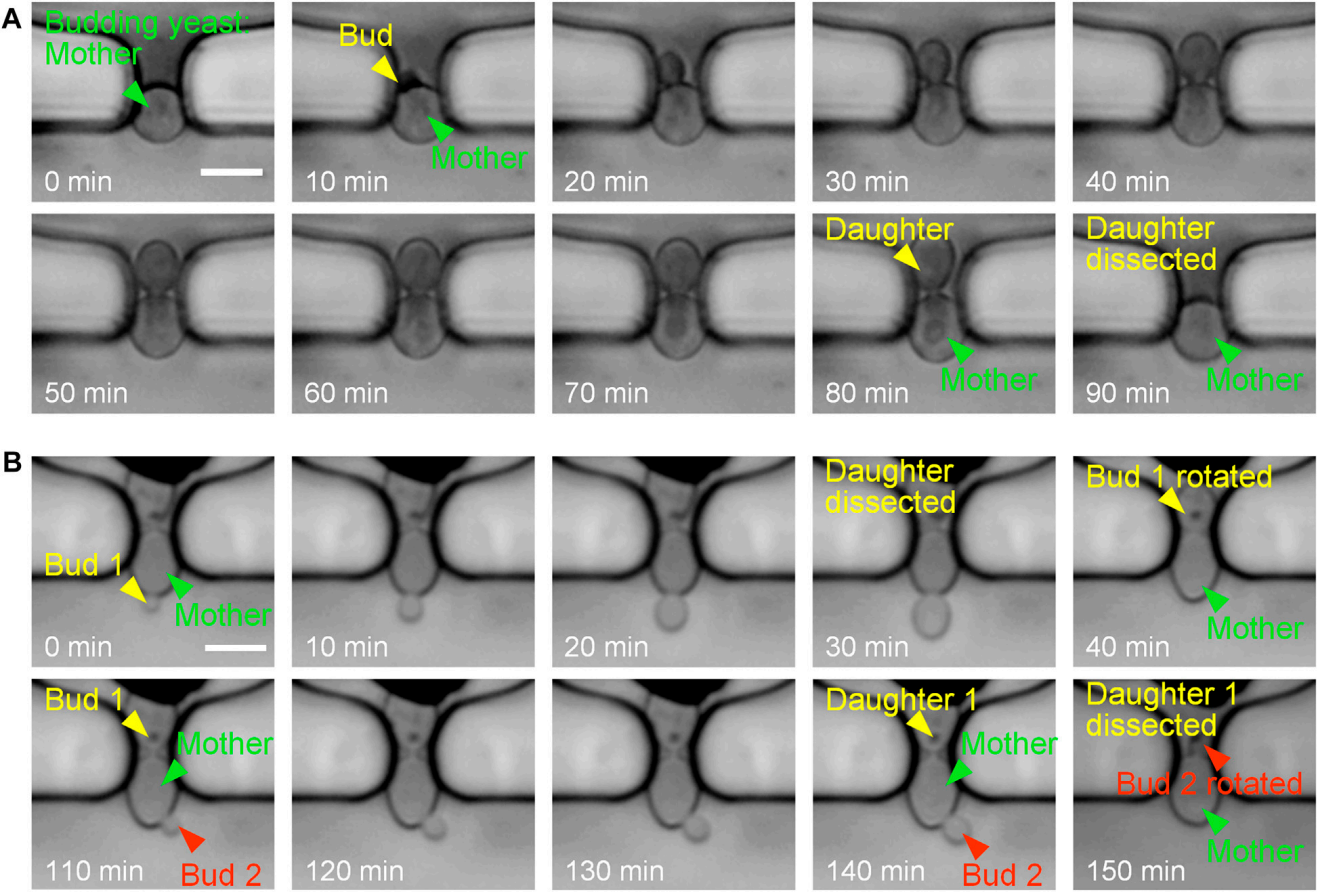
Time-lapse images at 10-min intervals of single budding yeast cells during continuous culturing. **(A)** Budding and daughter dissection of an immobilized yeast cell in one generation. **(B)** Budding and daughter dissections of an immobilized yeast cell in two generations with successful reorientation of newborn buds towards the side channel by flowing fluid. Scale bar is 4 μm.

Figure 3 shows the experimental results of single-cell trapping and continuous culturing. Single budding yeast cells were initially captured at the traps, and were reliably retained for bud growth and daughter dissection under the continuous perfusion of cell-culturing medium. Due to the size difference between yeast mother cells and buds, buds usually grew inside the trap or rotated into the trap for subsequent growth by hydrodynamic forces. After the completion of cell division and cytokinesis, daughter cells were ultimately dissected by fluid shear forces and washed away with the flowing medium. The automatic detachment of a yeast daughter cell under the hydraulic shear forces was known as a daughter-dissection event. For instance, the mother cell in Figure 3A sprouted at 10 min, its bud gradually grew to be a mature daughter, and finally the daughter cell was successfully removed at 90 min.

New buds of yeast cells may start to sprout when the previous daughter cells are not completely detached from mother cells in liquid culture. As shown in Figure 3B, both bud 1 and bud 2 started to grow when the former daughters were not dissected. After the mature progenies were automatically removed by the fluid flow (at 40 and 150 min), newborn buds were successfully rotated into the narrow orifice of the trap. In this case, cell rotation could be attributed to two reasons: i) the high flow velocity distributed in the narrow orifice exerting sufficient drag forces on buds from the main channel towards the side channel and ii) the high channel height providing adequate space in vertical direction for cell rotation. Therefore, the bud rotation and reorientation towards the side channel is of benefit to coincident and efficient daughter-cell dissections.

### Real-Time Monitoring of Yeast Dissection Events Using *In-situ* Electrical Impedance Biosensor

**FIGURE 4:**
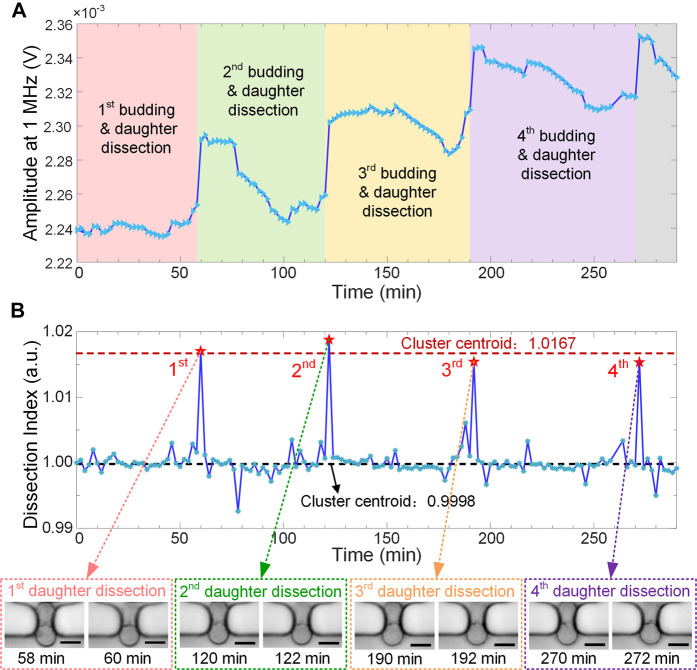
Real-time EIS monitoring of budding yeast growth and division with 4 daughter-dissection events in sequence. **(A)** Raw amplitude curve of EIS signals at 1 MHz along the recording time period. Sampling interval is 2 min **(B)** “*Dissection Index*” curve combined with K-means cluster analysis clearly showing 4 sharp spikes corresponding to daughter-dissection events of the recorded mother cell. Insets show micrographs of the yeast mother cell right before and after daughter removal. Scale bar is 4 μm.

During the continuous cell culturing, *in-situ* EIS measurement was performed every 2 min to monitor yeast budding process in a label-free and real-time manner. For the wide-band EIS, electric current at low frequencies (typically below 1 MHz) flows mostly around cells and very few penetrates cell membrane. Thus, EIS signal at 1 MHz can be directly used to characterize cell size or morphology, which has been validated previously (Zhu et al., 2015). In Figure 4A, raw signal amplitude at 1 MHz shows 4 remarkable jumps when 4 daughters were dissected and washed away from the traps in sequence. Such activities of daughter dissection cause abrupt reduction of cell size or volume, which can be sensitively recorded by the real-time EIS measurement.

The recorded raw EIS signal, referring to the instantaneous impedance of yeast cell and its surrounding medium, may exhibit drifts along the recording time period. They could be attributed to bud swing, cell reorientation, or dirt attachment in the sensing unit during cell culturing. To compensate signal drifts and accurately identify the momentary events of daughters separating from their mother, EIS amplitude at time point t , $A_{t}$ , was divided by the amplitude value at the previous time point, $A_{t-1}$ , thereby yielding a “*Dissection Index* (*DI*)” ( $DI=A_{t}/A_{t-1}$ ) curve (Figure 4B). During yeast budding, EIS signal varied slowly and slightly, resulting in the ratio *DI* approaching the baseline. Since the signal drifts were relatively low in amplitude, they were also normalized around the baseline. This simple normalization method could thus compensate potential drifts of EIS signal. In comparison, the sudden removal of a daughter from its immobilized mother cell at time point t caused the recorded signal amplitude $A_{t}$ to abruptly jump to a value much higher than that of $A_{t-1}$ , thus leading to a conspicuous peak on the *DI* curve. Moreover, by using K-means cluster analysis, data points on the *DI* curve were adaptively classified into two categories, i.e., the baseline cluster centered around 0.9998 with minor signal fluctuations, and the peak cluster centered around 1.0167 indicating the occurrence of daughter dissections. Therefore, the moments, when daughters were detached from the mother cell in sequence, were accurately extracted from the recorded EIS signals, and 4 daughter-dissection events were highlighted as 4 sharp spikes on the *DI* curve.

## Discussion

In this work, a microfluidic device integrated with EIS biosensor has been developed to perform real-time and *in-situ* impedance monitoring of daughter dissection events of budding yeast cells. In the microfluidic device, narrow bottleneck-like traps enable reliable retention of yeast mothers in single-cell resolution and provide high hydraulic shear forces for the automatic removal of daughters after the completion of cell division. The EIS sensing unit allows for label-free and sensitive detection of daughter dissections during continuous replication of budding yeast. “*Dissection Index* (*DI*)” minimizing signal fluctuations has been successfully used to compensate the EIS signal drifts derived from bud rotation, cell movement, or dirt attachment in the sensing unit. Associated with K-means cluster analysis, momentary events of daughters separating from their mother can be clearly identified from EIS signals.

With regard to present design of the microfluidic device, we have noticed a few problems or limitations on real-time monitoring of budding yeast replication and identification of daughter-dissection events by using the integrated electrical impedance biosensor. i) Although higher traps provide adequate space in vertical direction for cell rotation and reorientation, they increase the risk of stacking more cells in vertical space. Lower microfluidic channels and traps might reduce such risks, but would increase the hydraulic resistance in channels for cell delivery and cause channel clogging by big clusters of yeast cells. ii) Since the EIS sensing unit, especially the long common stimulus electrode, has much free space exposed in the fluidic channel, passage of cell suspension or capture of dirt may affect the measured impedance and give rise to noise or drift on the recorded EIS signal. iii) Current microfluidic device is limited to a throughput of 10 independent cell traps. Also, these traps feature non-uniform distribution of hydraulic pressure drops so that they cannot be simultaneously occupied by single cells under the same setting of medium flow rate and underpressure. As such, the present device is feasible to verify the concept of yeast-daughter-dissection monitoring and investigate the sensitivity of the integrated electrical impedance biosensor, but poses challenges to integrate more traps for high-throughput culturing and monitoring of single budding yeast cells. iv) Due to accidental cell loss or trapping of additional cell during the EIS recording, current version of the microfluidic device allows for only several-hour monitoring of cell growth and division in a single experiment, which cannot cover the whole replicative lifespan of budding yeast. The cell-culturing system, especially the microfluidic structure for single-cell trapping and long-term retention, needs to be optimized in terms of stability and robustness so that it can monitor single-cell replication over 48 h without interruption. Accordingly, the design of addressable electrodes to implement EIS measurement requires optimization to adapt for the new cell-culturing system.

To summarize, this proof-of-concept study has demonstrated that the EIS-integrated microfluidic device is capable of automatic removal of daughter cells by hydraulic shear forces and sensitive monitoring of daughter-dissection events by real-time EIS measurement. With further optimization and development in terms of long-term stability and throughput, the microdevice potentially provides an automatic and non-optical platform for yeast replicative aging studies and RLS determination.

## Data Availability

The original contributions presented in the study are included in the article/Supplementary Material, further inquiries can be directed to the corresponding authors.
